# Breast Tissue Composition and Its Dependence on Demographic Risk Factors for Breast Cancer: Non-Invasive Assessment by Time Domain Diffuse Optical Spectroscopy

**DOI:** 10.1371/journal.pone.0128941

**Published:** 2015-06-01

**Authors:** Paola Taroni, Giovanna Quarto, Antonio Pifferi, Francesca Abbate, Nicola Balestreri, Simona Menna, Enrico Cassano, Rinaldo Cubeddu

**Affiliations:** 1 Dipartimento di Fisica, Politecnico di Milano, Milano, Italy; 2 Istituto di Fotonica e Nanotecnologie, Consiglio Nazionale delle Ricerche, Milano, Italy; 3 European Institute of Oncology, Breast Imaging Unit, Milano, Italy; 4 European Institute of Oncology, Department of Radiology, Milano, Italy; University of Nebraska Medical Center, UNITED STATES

## Abstract

**Background:**

Breast tissue composition is recognized as a strong and independent risk factor for breast cancer. It is a heritable feature, but is also significantly affected by several other elements (e.g., age, menopause). Nowadays it is quantified by mammographic density, thus requiring the use of ionizing radiation. Optical techniques are absolutely non-invasive and have already proved effective in the investigation of biological tissues, as they are sensitive to tissue composition and structure.

**Methods:**

Time domain diffuse optical spectroscopy was performed at 7 wavelengths (635-1060 nm) on 200 subjects to derive their breast tissue composition (in terms of water, lipid and collagen content), blood parameters (total hemoglobin content and oxygen saturation level), and information on the microscopic structure (scattering amplitude and power). The dependence of all optically-derived parameters on age, menopausal status, body mass index, and use of oral contraceptives, and the correlation with mammographic density were investigated.

**Results:**

Younger age, premenopausal status, lower body mass index values, and use of oral contraceptives all correspond to significantly higher water, collagen and total hemoglobin content, and lower lipid content (always *p* < 0.05 and often *p* < 10^-4^), while oxygen saturation level and scattering parameters show significant dependence only on some conditions. Even when age-adjusted groups of subjects are compared, several optically derived parameters (and in particular always collagen and total hemoglobin content) remain significantly different.

**Conclusions:**

Time domain diffuse optical spectroscopy can probe non-invasively breast tissue composition and physiologic blood parameters, and provide information on tissue structure. The measurement is suitable for *in vivo* studies and monitoring of changes in breast tissue (e.g., with age, lifestyle, chemotherapy, etc.) and to gain insight into related processes, like the origin of cancer risk associated with breast density.

## Introduction

Breast tissue composition is essentially determined by the relative proportion of epithelial tissue, stroma and fat. It is a heritable feature, but it is also significantly affected by factors like age, pregnancy, and menopause [1,2,3,4]. Breast tissue composition is recognized as a strong and independent risk factor for breast cancer [5,6,7]. At present, it is generally quantified by mammographic density. Thus, its assessment involves the use of ionizing radiation.

Optical techniques, including both spectroscopy and imaging, are absolutely non-invasive and can be effectively applied *in vivo* to derive information on tissue composition and structure [8,9,10]. Over the years, most of the work performed in the optical field has been devoted to the detection of breast cancer and the monitoring of cerebral hemodynamics, and has focused on the assessment of oxy- and deoxyhemoglobin content or on their relative variations. However, some research groups have successfully developed and applied optical instruments that allow also the quantification of water and lipids in breast tissue [11][12] and of scattering parameters that are related to the overall structure of tissue at microscopic level [13][14].

Our group has longed worked on the development of advanced optical systems and their application for the non-invasive characterization of biological tissues [15,16,17,18]. The time domain approach we adopt consists in injecting short (picosecond) light pulses into the tissue and measuring the pulses that are re-emitted after propagation through the tissue. The shape of the re-emitted pulse depends on the optical properties of the medium. Thus, its analysis allows one to perform a complete optical characterization of the medium with the independent assessment of both the absorption and scattering coefficients. When time domain measurements are carried out (and the absorption properties are consequently measured) at several wavelengths, then tissue composition can be estimated from the Beer law. For a more complete and accurate tissue characterization, we have progressively extended the spectral range of operation of our instruments, which allowed us to quantify *in vivo* not only blood parameters, water and lipids, but also collagen [19]. Moreover, information on tissue structure at a microscopic level is obtained from the scattering parameters (amplitude and power). In fact, a simple empirical approximation to Mie theory relates them to the concentration and equivalent size of scattering centers (cells, nuclei, and organelles) in tissue [20][21].

Previously, we have combined these pieces of information specifically aiming at the non-invasive optical assessment of breast density and identification of subjects that are at high risk for developing breast cancer because of their high breast density [22][23].

More in general, in the present work we show how time domain diffuse optical spectroscopy can effectively be used to study the dependence of breast tissue composition (water, lipid and collagen concentrations, total hemoglobin content and oxygen saturation level) and structure (scattering amplitude and power) on various demographic parameters (age, menopausal status, body mass index, use of oral contraceptives).

The availability of a fully non-invasive means to provide quantitative information on tissue composition and monitor its changes could be exploited to achieve a better understanding of breast physiology and of the biological basis of breast cancer, and could contribute to foster the development of personalized medicine procedures.

## Methods

### Instrument set-up

Our portable clinical instrument for time-resolved optical mammography operates in transmittance geometry, *i*.*e*., in the same geometry as x-ray mammography.

Seven pulsed diode lasers (LDH-P-XXX, PicoQuant, Germany, where XXX represents the nominal wavelength in nanometers) are used as light sources emitting at 635, 685, 785, 905, 930, 975 and 1060 nm, with average output powers of ~1–5 mW, temporal widths of ~150–400 ps (full width at half maximum, FWHM), and repetition rates of 20 MHz. A single driver (PDL-808 “Sepia”, PicoQuant, Germany) controls all the laser heads, and their output pulses are properly delayed by means of graded index optical fibers, and combined into a single coupler. Circular variable neutral density filters in each of the 7 illumination paths allow optimization of the illumination power at each wavelength separately. The breast is softly compressed between parallel antireflection-coated tempered glass plates. A fiber bundle collects the output light on the opposite side of the compression unit. The distal end of the bundle is bifurcated, and its two legs guide photons respectively to a photomultiplier tube (PMT) for the detection of wavelengths <850 nm (R5900U-01-L16, Hamamatsu, Japan) and to a PMT for longer wavelengths (H7422P-60, Hamamatsu, Japan). Circular variable neutral density filters, placed in front of each PMT, are used to control the illumination power during *in vivo* measurements and for the acquisition of the instrument response function. In particular, for *in vivo* measurements the attenuators are automatically rotated by computer-controlled stepper motors to reach a pre-set number of counts (2 x 10^5^ counts/s) at each wavelength in a reference position (*i*.*e*. close to the chest wall). Two personal computer (PC) boards for time-correlated single photon counting (SPC134, Becker&Hickl, Germany) allow the acquisition of time-resolved transmittance curves at wavelengths shorter and longer than 850 nm, respectively. Depending on the wavelength, the instrument response function ranges between 460 and 930 ps (FWHM).

The illumination fiber and collecting bundle are scanned in tandem and a feedback on the total number of counts per point, ruled by an adjustable threshold, restricts the scan to the breast area. To minimize dead times, continuous acquisition is performed and data are stored every millimeter of path, *i*.*e*. every 25 ms. The scan time depends on the area of the compressed breast that needs to be imaged, but is typically around 5 min. Notwithstanding measurement times significantly longer than required by x-ray mammography, the great majority of the subjects who participated in the study did not report any significant discomfort, and overall they judged the optical measurement more acceptable than x-ray mammography. When some complaint was made, it was on the still position that could cause discomfort to the back more than on the compression itself.

The compression unit can be rotated by an angle up to 90° in both clock-wise and counter-clock-wise direction, so that images of both breasts can be recorded in the cranio-caudal (CC) as well as medio-lateral or oblique (OB, at 45°) views.

The entire set-up is a stand-alone instrument (50 cm W x 80 cm D x 140 cm H), mounted on wheels and approved by the Italian Ministry of Health for use in a clinical environment. Details on the instrument set-up and performances, and on the procedures for data acquisition and analysis are reported in [24].

### Ethics statement

Written informed consent was obtained from all subjects and all protocols were approved by the Institutional Review Board of the European Institute of Oncology (study n°. S305/306).

### Subjects and measurement procedure

Data were collected between 2009 and 2013 from 200 subjects as part of a study on the optical characterization of malignant and benign breast lesions and the optical assessment of breast tissue composition and structure for the non-invasive estimate of breast density. Ninety-five subjects had a malignant lesion and 27 had a benign lesion. Demographic information on the subjects involved in the study is reported in **Table 1** and **S1 File**.

**Table 1:** Subject demographics^a^ (*N* = 200).

Age (y)	52.2± 11.7 (30.0–79)
Height (cm)	162 ± 6 (147–180)
Weight (kg)	62.4 ± 11.1 (43.0–107)
BMI (kg/m^2^)	23.6 ± 3.8 (16.5–35.5)
Premenopausal status	*N* = 93^b^
Postmenopausal status	*N* = 102
Use of OCs	*N* = 88^b^
No use of OCs	*N* = 110

^a^Average and standard deviation (range) of continuous variables and number *N* of subjects in a specific condition for categorical variables.

^b^Information was not available for *N* = 5 subjects on menopausal status and for *N* = 2 subjects on the use of OCs.

From each subject, four sets of 7-wavelength transmittance images were acquired (*i*.*e*. CC and OB views of both breasts). For the entire duration of the acquisition, the subject was sitting comfortably.

Recent (less than 1 year old) x-ray mammograms of the same views were generally available or were acquired in the same measurement session as optical images.

### Data analysis

#### Optical data

Concerning optical data, light propagation in breast tissue was modeled using the diffusion approximation to the radiative transport theory with extrapolated boundary condition, for a homogeneous infinite slab [25][26]. This allowed us to estimate the optical properties (absorption and reduced scattering coefficient, *μ*_*a*_ and *μ'*_*s*_, respectively) at 7 wavelengths. Information on tissue composition and structure were obtained directly from time-resolved transmittance curves measured at the 7 wavelengths. The Beer law was used to relate the absorption properties *μ*_*a*_ *(λ)* at the wavelength *λ* to the concentrations *c*_*i*_ of the main tissue constituents:

$$\mu_{a}\left(\lambda \right)=\sum_{i}c_{i}\varepsilon_{i}\left(\lambda \right),$$ (1)

where *ε*_*i*_ *(λ)* is extinction coefficient of the *i*-th constituent at the wavelength *λ*. The scattering properties were modeled through a simple approximation to Mie theory:

$$\mu_{s}^{'}\left(\lambda \right)=a{\left(\frac{\lambda }{\lambda_{o}}\right)}^{-b},$$ (2)

where *λ*_*o*_ = 600 nm,*a* is the reduced scattering coefficient *μ’*_*s*_ *(λ*_*o*_ *)*, and *b* is the scattering power, which describes the slope of the scattering spectrum [20][21]. A spectrally constrained global fitting procedure was applied that provides robust results even when the data are collected at a limited number of wavelengths [27]. Free parameters of the fit were the concentrations of oxy- and deoxy-hemoglobin (*HbO*_*2*_ and *Hb*, respectively), water, lipids, and collagen, together with the scattering amplitude *a* and power *b*. From *Hb* and HbO_2_, the total hemoglobin content *tHb = Hb + HbO*_*2*_ and the oxygen saturation level *SO*_*2*_ = *HbO*_*2*_ */tHb* were then calculated.

For each image pixel (1 mm^2^), this procedure provided optical properties, tissue composition and information on microscopic structure averaged along the line of site between injection and collection points. Then, for each subject all data from the four images (CC and OB views of both breasts) were averaged to provide the average characterization of the breast tissue of that subject (**S1 File**). Regions of marked inhomogeneity (as observed in the case of some breast lesions and close to the boundaries of the compressed breast that may be affected by measurement artefacts) were excluded from the average [24].

#### X-ray mammographic images

When available (*N* = 189), recent x-ray mammograms were analyzed to assess the mammographic density. An expert radiologist (FA) assigned Breast Imaging and Reporting Data System (BI-RADS) mammographic density categories as: i) almost entirely fat (category 1, *N* = 27); ii) scattered fibroglandular densities (category 2, *N* = 55); iii) heterogeneously dense (category 3, *N* = 70); and iv) extremely dense (category 4, *N* = 37) [28].

#### Statistical analysis

The statistical significance of the difference between two groups (*e*.*g*., water content in premenopausal *vs*. postmenopausal subjects) was assessed using the Mann-Whitney test at alpha = 0.05. Significance was considered when *p* < 0.05.

When regression was adopted to model the relationship between continuous variables (*e*.*g*., water content *vs*. age), the adjusted *R* ^*2*^ value was used to estimate the percentage of the variation of the dependent variable that can be accounted for by the regression model.

The linear correlation between blood parameters (either *tHb* or *SO*_*2*_) and lipid content in pre- and post-menopausal women was appraised through the Pearson correlation coefficient *r*.

Statistical analysis was performed using IBM SPSS Statistics Release 21 and Minitab Version 16.

## Results and Discussion

### Breast density

**Fig 1:**
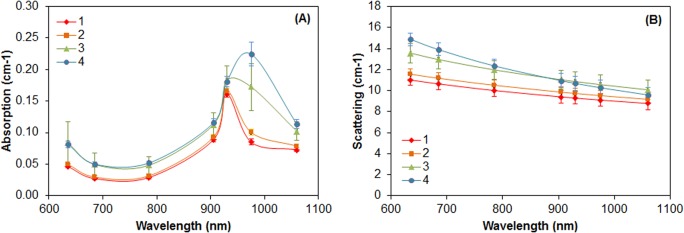
Average absorption and reduced scattering spectra of 189 subjects classified in different BI-RADS categories. **A)** Absorption spectra and **B)** reduced scattering spectra averaged over *N* = 27 subjects in Bi-RADS category 1 (red diamonds), *N* = 55 subjects in category 2 (orange squares), *N* = 70 subjects in category 3 (green triangles), and *N* = 37 in category 4 (blue circles). The error bars show the standard deviation of the absorption (A) or reduced scattering (B) values calculated on all subjects that belong to the same BI-RADS category.

The optical properties (both absorption and scattering) of breast tissue reflect its composition and consequently show marked inter-subject variation. Mammographic density is generally used as a measure of breast tissue composition [28]. Thus the easiest way to investigate inter-subject differences is to compare the optical properties of breasts belonging to different BI-RADS categories, spanning from almost entirely fat (category 1) to extremely dense (category 4). **Fig 1** shows the average absorption and scattering spectra corresponding to distinct BI-RADS categories. The major differences in breast tissue composition corresponding to different mammographic categories are already evident when looking at the absorption spectra. In particular, the spectrum of dense breasts is dominated by the absorption of water, that *in vivo* is expected to peak around 970 nm, while the sharper peak of lipids centered around 930 nm stands out in the case of adipose breasts. The higher collagen content in tissue with a high fibroglandular fraction is less apparent, because in this spectral range the protein is characterized by weak absorption as compared to the other major constituents. However, it still contributes non-negligibly to the higher absorption of dense breasts at the longest wavelength (1060 nm).

Differences are observed also in the scattering properties (**Fig 1B**). Stroma and epithelium are abundant in dense breasts. As compared to adipose tissue, they are characterized by higher scattering, decreasing with steeper slope as a function of wavelength. This indicates that in dense tissue there is a higher number of “scattering centers”, namely interfaces that alter the direction of propagation of photons at microscopic level, causing light diffusion (higher amplitude *a*). Moreover, these centers have smaller average size (higher scattering power *b*).

Data fitting to the diffusion theory allows us to derive quantitative information on the different properties of tissue of different mammographic density, as shown in **Table 2**. Upon increasing mammographic density from BI-RADS category 1 to 4, all optically derived parameters except *SO*_*2*_ gradually change, in agreement with the expected change in tissue composition that determines the progressively higher attenuation of x-rays leading to higher BI-RADS reading. In particular, water and collagen, major constituents of dense stroma, increase while lipids decrease. Correspondingly, both scattering parameters (*a* and *b*) gradually grow. Increased vascularization and metabolism could be expected in dense tissue, leading to higher total hemoglobin content *tHb* and lower oxygen saturation *SO*_*2*_. Based on optical outcomes, the former hypothesis is confirmed, while the latter is only supported by the decreasing average values, while no statistical difference is observed when adjacent BIRADS categories are compared (**Table 2**).

**Table 2:** Tissue composition and scattering parameters of subjects in different BI-RADS categories.

BI-RADS	Water^a^ (mg/cm^3^)	Lipid^a^ (mg/cm^3^)	Collagen^a^ (mg/cm^3^)	tHb^a^ (μM)	SO_2_ ^a^ (%)	a^a^ (cm^-1^)	b^a^ (-)
**1** (*N* = 27)	75.01 (14.89)	775.10 (43.82)	44.94 (18.20)	8.76 (1.49)	89.83 (6.52)	11.40 (0.81)	0.43 (0.10)
	[0.0037]^b^	-	-	-	-	-	-
**2** (*N* = 55)	97.76 (53.99)	739.16 (75.53)	50.90 (21.06)	9.27 (3.16)	89.19 (8.10)	12.00 (2.30)	0.48 (0.15)
	[<10^–4^]^b^	[<10^–4^]^b^	[<10^–4^]^b^	[0.0028]^b^	-	[0.0003]^b^	[0.0001]^b^
**3** (*N* = 70)	193.26 (111.07)	647.85 (112.24)	67.23 (44.99)	10.85 (2.81)	87.22 (10.23)	14.04 (3.36)	0.62 (0.24)
	[0.0001]^b^	[<10^–4^]^b^	[0.0112]^b^	[0.0493]^b^	-	[0.0049]^b^	[<10^–4^]^b^
**4** (*N* = 37)	330.91 (167.43)	531.70 (119.00)	113.80 (34.07)	14.86 (4.22)	86.68 (10.90)	15.12 (2.24)	1.05 (0.292)

^a^Average values and standard deviation (in round brackets).

^b^In square brackets, *p*-value (Mann-Whitney test) for the difference between the mean values reported in the above and below cells (*e*.*g*., water content for subjects in BI-RADS category 1 *vs* 2). Reported only when significant (*p* < 0.05).

### Age

Increasing age generally determines a progressive decrease in mammographic density, explained with a change in the composition of breast tissue, where translucent adipose tissue progressively replaces the radio-opaque epithelial and stromal components [29]-[30][31].

**Fig 2:**
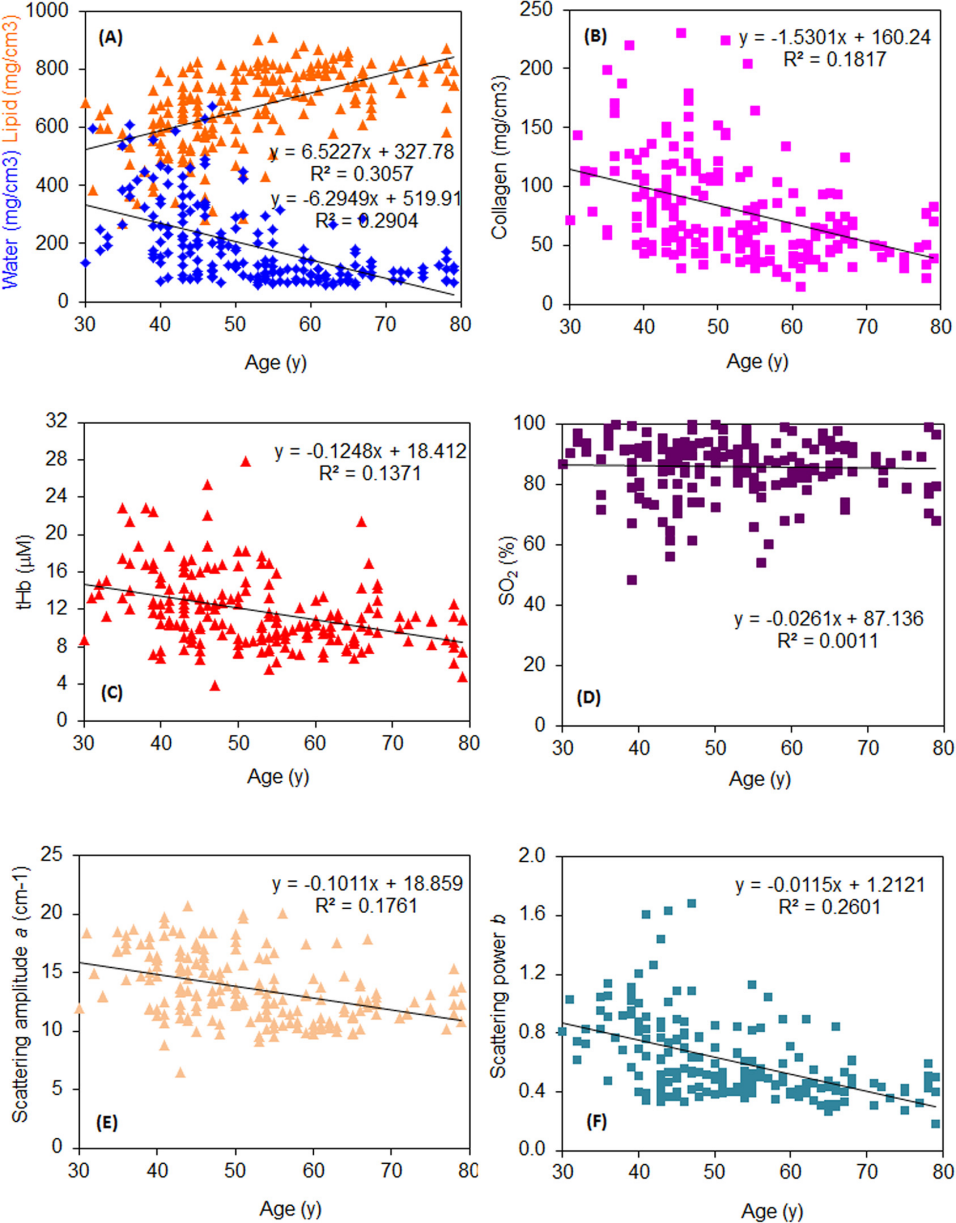
Dependence of optically derived tissue parameters on age. **A)** water (blue) and lipid (orange), **B)** collagen, **C)** total hemoglobin content *tHb*, **D)** oxygen saturation *SO*_*2*_, **E)** scattering amplitude *a*, and **F)** scattering power *b*.

Data reported in the literature show that the change is evident upon increasing age up to approximately 50 y, while no clear trend of variation seems to be present thereafter [32]. As shown in **Fig 2A and 2B**, data on optically derived tissue composition upon increasing age are spread, but they confirm some decrease in water and collagen content (fibroglandular fraction of tissue) and corresponding increase in the lipid content (adipose fraction). Some correspondence with the reported dependence of mammographic density on age [32] is confirmed by the fact that the quadratic regression of an optically derived tissue constituent (water, lipids or collagen) versus age performs better than the linear one, even though only slightly (*e*.*g*., *R* ^*2*^ = 32.7% for the quadratic regression of water *vs*. age, and *R* ^*2*^ = 28.7% for the linear regression). Alternatively, the comparison of the linear regression performed on data for age ≤ 50 y and > 50 y could be considered. As an example, in the case of water, the fitting lines are Y = -9.623X + 672.2 (r = 0.31) for the former group and Y = -2.036X + 245.8 (r = 0.23) for the latter one, confirming a much clearer decrease in younger women (≤ 50 y).

More in detail, as displayed in **Table 3**, on average the breasts of younger women (≤50 y) are characterized by significantly higher water and collagen content than the breasts of older ones (> 50 y) while the opposite trend is observed for the lipid content. The total hemoglobin content *tHb* and both scattering parameters are also significantly higher in younger women, in agreement with the higher weight of the fibroglandular fraction (water and collagen).

**Table 3:** Tissue composition and scattering parameters of subjects ≤ 50 y and > 50 y.

Age	Water^a^ (mg/cm^3^)	Lipid^a^ (mg/cm^3^)	Collagen^a^ (mg/cm^3^)	tHb^a^ (μM)	SO_2_ ^a^ (%)	a^a^ (cm^-1^)	b^a^ (-)
≤ **50 y** (*N* = 101)	261.08 (148.48)	595.45 (129.29)	95.76 (42.32)	13.16 (4.04)	85.48 (10.02)	14.76 (2.74)	0.73 (0.29)
	[<10^–4^]^b^	[<10^–4^]^b^	[<10^–4^]^b^	[<10^–4^]^b^	-	[<10^–4^]^b^	[<10^–4^]^b^
> **50 y** (*N* = 99)	119.62 (72.25)	743.08 (102.24)	64.53 (35.37)	10.60 (3.39)	86.07 (8.54)	12.37 (2.36)	0.49 (0.16)

^a^Average values and standard deviation (in round brackets).

^b^In square brackets, *p*-value (Mann-Whitney test) for the difference between the mean values reported in the above and below cells. Reported only when significant (*p* < 0.05).

It is interesting to observe that the relative difference in the amount of collagen between younger and older women, measured here *in vivo* non-invasively, agrees very well with what reported in the literature for the quantification of collagen performed on 3-μm thick histologic sections obtained from women in the same age categories [33].

When the whole population is considered, the total hemoglobin content *tHb* decreases slightly with age, while the oxygenation level *SO*_*2*_ does not show any significant dependence on age (**Fig 2C and 2D**). A slight decrease with age is also observed for both scattering amplitude *a* and power *b*, as expected based on the increasing lipid content (**Fig 2E and 2F**).

### Menopausal status

Menopause affects the composition of breast tissue even more markedly than age, leading to a significant replacement of stromal and epithelial tissue by fat. Our results fully agree with these observations, based on mammography and magnetic resonance imaging (MRI) [31],[34]. As shown in **Table 4**, on average the breast has higher water and collagen content, and lower lipid content before menopause than after it. Correspondingly, in premenopausal women breast tissue scattering is more marked (higher *a*, suggesting higher density of scattering centers) and the scattering spectra are steeper (higher *b*, indicating smaller equivalent size of the scattering centers), as expected when the ratio of fibrogladular tissue to adipose tissue is higher. The total hemoglobin content *tHb* is also markedly higher before menopause than after it.

**Table 4:** Tissue composition and scattering parameters of pre- and postmenopausal subjects.

	Water^a^ (mg/cm^3^)	Lipid^a^ (mg/cm^3^)	Collagen^a^ (mg/cm^3^)	tHb^a^ (μM)	SO_2_ ^a^ (%)	a^a^ (cm^-1^)	b^a^ (-)
**Pre-menopausal** (*N* = 93)	263.05 (148.36)	598.29 (133.48)	98.73 (42.97)	13.64 (4.06)	86.82 (9.64)	14.85 (2.60)	0.70 (0.27)
	[<10^–4^]^b^	[<10^–4^]^b^	[<10^–4^]^b^	[<10^–4^]^b^	[0.02]^b^	[<10^–4^]^b^	[<10^–4^]^b^
**Post-menopausal** (*N* = 102)	127.92 (86.30)	729.998 (111.17)	64.25 (34.11)	10.39 (3.21)	84.87 (9.10)	12.44 (2.52)	0.53 (0.24)

^a^Average values and standard deviation (in round brackets).

^b^In square brackets, *p*-value (Mann-Whitney test) for the difference between the mean values reported in the above and below cells.

All these differences between pre- and postmenopausal women closely resemble what is described above for the difference between women aged ≤50 y and >50 y. However, interestingly the menopausal status seems to affect also the blood oxygenation level that is significantly higher in premenopausal women than in postmenopausal ones, even though with lower significance than all other parameters (*p* < 0.02 instead of *p* < 10^–4^).

The reduced estrogen levels after menopause is expected to lower the metabolic rate. However, it is not immediately obvious how this could lead to a reduced hemoglobin saturation. Studies performed on small animals have shown that a marked increase in body fat is not accompanied by a corresponding increase in vascularization, and this leads to hypoxia of the adipose tissue in obese subjects [35]. Thus, lower levels of hemoglobin oxygenation in post-menopause women might be related to the significant increase in the adipose fraction of breast tissue that occurs with menopause. We detect negative correlation between *tHb* and lipid content for both pre-menopausal women (Pearson correlation coefficient *r* = -0.402, *p* < 0.001) and post-menopausal women (*r* = -0.503, *p* < 0.001), while no significant correlation is observed between *SO*_*2*_ and lipid content. However, this does not rule out the existence of a non-linear relationship.

It is important to observe that the menopausal status has stronger effect on breast tissue than just age [31]. In fact, if age-matched subjects are considered, all optically derived parameters are still significantly different (*p* < 0.01) before versus after menopause, except for the blood oxygenation level and the scattering slope.

### Body Mass Index (BMI)

**Fig 3:**
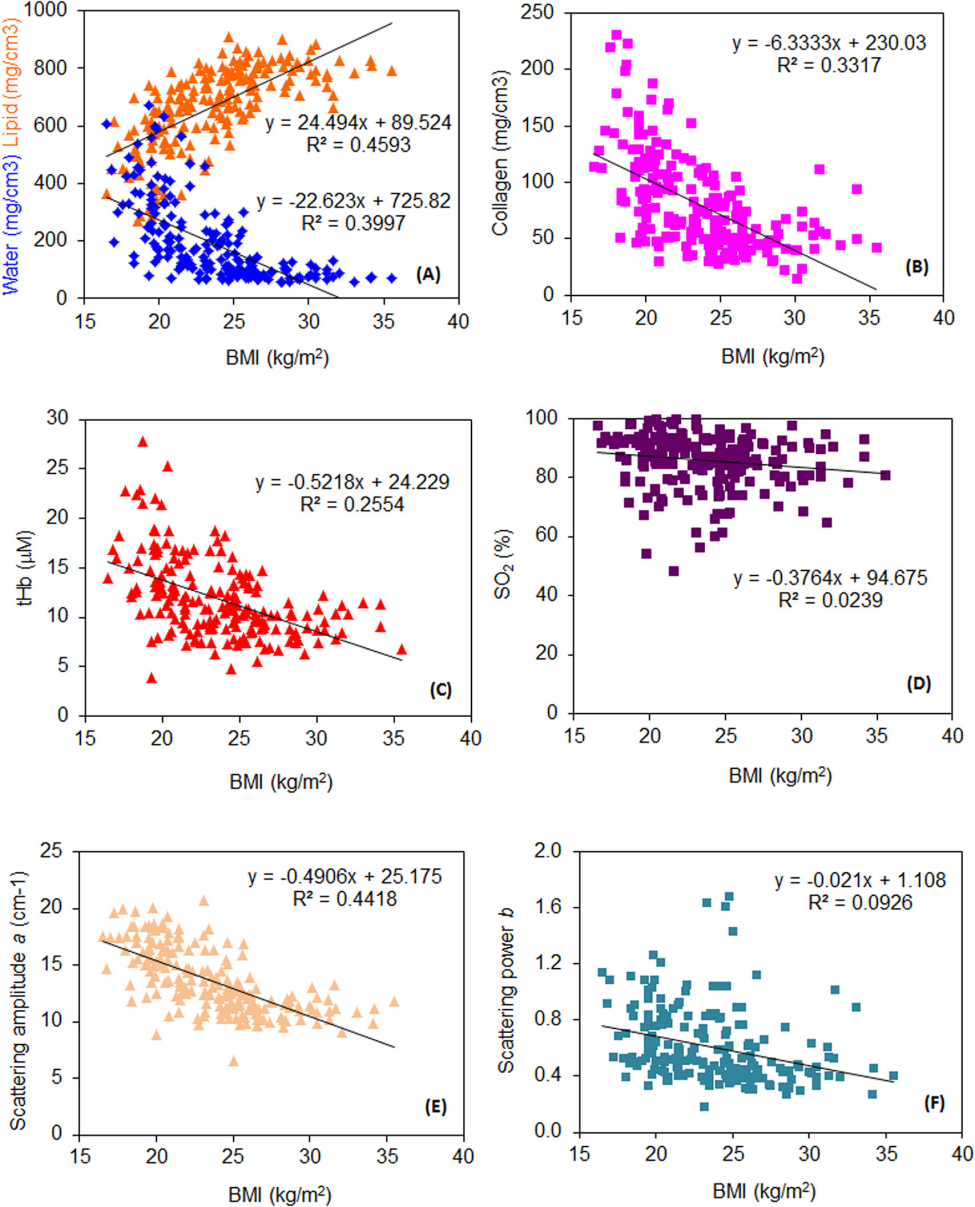
Dependence of optically derived tissue parameters on BMI. **A)** water (blue) and lipid (orange), **B)** collagen, **C)** total hemoglobin content *tHb*, **D)** oxygen saturation *SO*_*2*_, **E)** scattering amplitude *a*, and **F)** scattering power *b*.

As displayed in **Fig 3A and 3B**, for increasing values of BMI a progressive change in breast tissue composition is observed: the amount of lipids increases, while the amounts of water and collagen decrease, to imply a higher weight of the adipose tissue fraction in the breast of women with higher BMI. These observations are in agreement with dependence on BMI of dense breast volume measured by non-contrast MRI [36].

Even though the correlation is weaker than for BMI, the amount of lipids increases as a function of weight, while the amounts of water and collagen decrease. The opposite trend is observed versus height. These observations (data not shown) are again in agreement with measurements performed with MRI [36][37].

For both blood parameters, the situation is similar to what observed versus age: slight decrease in *tHb* and no significant change in *SO*_*2*_ (**Fig 3C and 3D**).

The scattering amplitude also shows significant negative correlation with BMI, while the dependence of the scattering power seems to be much weaker (**Fig 3E and 3F**). As already observed for the dependence on age, a decrease in scattering amplitude and power agrees with the observed changes in tissue composition that indicate an increase of the adipose component upon increasing BMI.

### Use of Oral Contraceptives (OCs)

For all subjects but two, it was known if they had ever made use of OCs, while for most of them complete information on the use of OCs (present or past use, duration of use, etc.) was not available. So, only two groups were considered: women who had never used OCs and women who had used OCs in the past or were still using them at the time of the optical measurements.

**Table 5** compares optically derived parameters obtained on women who used OCs and who did not use them. Tissue composition (water, lipid and collagen content) and blood parameters (*tHb* and *SO*_*2*_) are all significantly different between the two groups, indicating higher water and collagen content, blood volume and oxygenation level, and lower lipid content in women who used OCs.

**Table 5:** Tissue composition and scattering parameters of subjects who used and who did not use OCs.

	Water^a^ (mg/cm^3^)	Lipid^a^ (mg/cm^3^)	Collagen^a^ (mg/cm^3^)	tHb^a^ (μM)	SO_2_ ^a^ (%)	a^a^ (cm^-1^)	b^a^ (-)
**Use of OCs** (*N* = 88)	166.58 (143.51)	664.99 (134.80)	86.67 (44.30)	12.14 (4.16)	89.77 (8.37)	13.49 (2.92)	0.53 (0.27)
	[0.0217]^b^	[0.0043]^b^	[0.0004]^b^	[0.0139]^b^	[0.0099]^b^	-	-
**No use of OCs** (*N* = 110)	120.45 (129.81)	725.30 (137.61)	60.28 (38.17)	10.71 (3.71)	85.71 (9.88)	12.68 (2.71)	0.51 (0.26)

^a^Average values and standard deviation (in round brackets).

^b^In square brackets, *p*-value (Mann-Whitney test) for the difference between the mean values reported in the above and below cells. Reported only when significant (*p* < 0.05).

However, it has to be taken into account that women who used OCs were significantly younger (47.7 ± 8.9 y *vs*. 55.5 ± 12.3 y) than other women. Based on what described above for the dependence of optically derived parameters on age, this could at least in part explain the observed differences between the two groups. Moreover, the group of “OC users” include both present and past users. So an attempt was made to identify and compare more homogeneous groups.

For this purpose, we considered subjects in the age range 31–75 y (*N* = 88 for OC users and *N* = 101 for non-users). We further divided the age range in 5-y wide sub-ranges (31–35 y, 36–40 y, etc.). We estimated the average values of all optically derived parameters within each sub-range. If the resulting distributions are compared using the paired Student’s t-test, none of the parameters is significantly different between OC users and non-users. However, the difference is close to statistical significance for *SO*_*2*_ (*p* = 0.0594) and collagen content (*p* = 0.0761).

As mentioned above, the group of OC users include both past users and current users at the time of the optical measurements. Unfortunately, only a small group of subjects (*N* = 12) are known to have been current OC users when the study was performed. Their age ranges between 30 and 54 y (average age 41.9 ± 7.4 y). In order to observe the effects of OC use independent of age, we compared the group of current OC users with subjects who had never used OCs and belong to the same age range (*i*.*e*., 30–54 y). The latter group include *N* = 54 subjects with average age 45.2 ± 5.7 y. The age distributions of the two groups are not significantly different (*p* = 0.106).

When the two groups are compared, on average the trend for tissue composition and blood parameters is the same as already observed on the full dataset (Table 5), with OC users characterized by higher water, collagen, *tHb* and *SO*_*2*_, and by lower lipid content. However, the differences are significant only for *tHb* (*p* = 0.0192).

Positive correlation was reported between OC use and breast epithelial proliferation in normal breast tissue [38]. Increased proliferation may increase the risk of neoplastic transformation, leading to the well-known increased breast cancer risk observed in OC users [39]. Optical measurements have recently allowed to detect a positive correlation between hemoglobin content in breast cancer tissue and mitotic count score, considered as a proliferative marker [40]. The higher *tHb* content we observe in OC users might thus be related to OC-induced proliferation of breast tissue.

Women often report breast tenderness as a side effect of OC use, suggesting that OC use might lead to an increase in breast water content. However, in univariate analysis per cent water and total breast water resulted not to be associated with current OC use [37]. As mentioned above, the information on OC use at the time of our optical measurements is available only for *N* = 12 subjects. This small group of current OC users on average is characterized by somehow higher water content than *N* = 54 subjects who are in the same age range and have never used OCs, but the difference is clearly not significant (224.72 ± 150.67 mg/cm^3^ *vs*. 197.03 ± 150.39 mg/cm^3^, *p* = 0.3729).

It is interesting to note that the mammographic density as assessed by BI-RADS categories was not significantly different for OC users *vs*. non-users. Moreover, the higher collagen content we observe in breast tissue of OC users (even though the difference is only close to statistical significance) seems not to agree with the negative correlation between absolute area of dense breast tissue and years of OC use that has been reported in the literature [2]. However, the same study did not detect any correlation of the non-dense area with years of OC use. Furthermore, we estimate the amount of collagen per unit volume of breast tissue (mg/cm^3^). So, correlation should be more likely occur between collagen content and percentage dense area than between collagen content and absolute dense area, and the two values (percentage area and absolute area) not necessarily show strong positive association. Thus, the comparison between our results and literature data is not straightforward.

## Conclusions

We have investigated by non-invasive optical means the dependence of breast tissue composition (water, lipid and collagen content), blood parameters (total blood volume and oxygen saturation level), and tissue structure (derived from scattering parameters) on breast density, age, menopausal status, BMI and use of OCs in a group of 200 subjects.

It is important to remind that the optical technique proposed here is safe (no ionizing radiation), practical, repeatable, relatively inexpensive, with good patient acceptance.

As observed by studying OCs users *vs* non-users, time domain optical measurements can provide insight into tissue conditions and changes that goes beyond what typically obtained from x-ray imaging. Optical means yield separate quantitative information on distinct tissue constituents (and structural parameters). On the contrary, in the case of mammography all these quantities contribute to determine a single variable, the x-ray attenuation, where differences in individual parameters may average out with consequent loss of detectable information.

Breast density depends strongly on tissue composition, which in turn is affected by several parameters, including age, menopausal status, BMI and use of OCs. Optical probing can be used to investigate these relationships *in vivo* to better understand them and to gain insight into the related breast cancer risk.

In particular, the estimate of collagen content *in vivo* in breast tissue might also prove useful for a more direct estimate of cancer risk than provided by the mammographic assessment of breast density. In fact, collagen is directly involved in cancer development and progression, and collagen in breast tumor tissue showed to differ from collagen in healthy tissue, both qualitatively and quantitatively [41,42,43]. Moreover, collagen is key to determine tissue stiffness that has recently proved to be significantly associated with breast cancer risk [44].

Due to the absolute non-invasiveness of the optical technique, young women could also be monitored gaining useful information on risk prediction and potentially designing dedicated preventive interventions for subjects that are at high risk.

It has also been suggested that changes in breast density (and consequently in tissue composition) may be useful in predicting response to chemopreventive treatments aimed at blocking breast cell proliferation [45].

Moreover, several studies have been published in the last years showing promises of optical techniques for effective monitoring and even prediction of the response to neoadjuvant chemotherapy, and systematic work is now on-going [46][47]. Changes associated with response have been reported in hemoglobin content and oxygenation level, as most of the optical instruments available for clinical use focus of the assessment of blood parameters. However, in some cases, also water or lipid content and scattering power were estimated and proved to correlate with therapeutic response. On the other side, breast stromal enhancement on MRI was shown to be associated with response to therapy [48], tissue stiffness estimated by shear-wave elastography showed promise for response prediction [49], and changes in mammographic breast density seem predictive of response to adjuvant endocrine therapy [50]. All these results suggest that monitoring and predicting the response to neoadjuvant chemotherapy may take advantage of a more thorough estimate of tissue composition and structure, including in particular collagen that plays major role in determining breast stromal enhancement on MRI, tissue stiffness, and mammographic density.

## Supporting Information

S1 File Dataset. Demographic information and optically derived parameters.
(PDF)

## References

[1] Ghosh K, Brandt KR, Reynolds C, Scott CG, Pankratz VS, Riehle DL, et al Tissue composition of mammographically dense and non-dense breast tissue. Breast Cancer Res Treat. 2012;131: 267–75. 10.1007/s10549-011-1727-4 21877142 PMC3707294

[2] Stone J, Dite GS, Gunasekara A, English DR, McCredie MRE, Giles GG, et al The heritability of mammographically dense and nondense breast tissue. Cancer Epidemiol Biomarkers Prev. 2006;15: 612–7. 10.1158/1055-9965.EPI-05-0127 16614099

[3] Boyd NF, Dite GS, Stone J, Gunasekara A, English DR, Mccredie MRE, et al Heritability of mammographic density, a risk factor for breast cancer. N Engl J Med. 2002;347: 886–894. 12239257 10.1056/NEJMoa013390

[4] Boyd NF, Martin LJ, Bronskill M, Yaffe MJ, Duric N, Minkin S. Breast tissue composition and susceptibility to breast cancer. J Natl Cancer Inst. 2010;102: 1224–37. 10.1093/jnci/djq239 20616353 PMC2923218

[5] Mccormack VA, Silva S. Breast Density and Parenchymal Patterns as Markers of Breast Cancer Risk : A Meta-analysis. Cancer Epidemiol Biomarkers Prev. 2006;15: 1159–1169. 10.1158/1055-9965.EPI-06-0034 16775176

[6] Boyd NF, Martin LJ, Yaffe MJ, Minkin S. Mammographic density and breast cancer risk: current understanding and future prospects. Breast Cancer Res. 2011;13: 223 10.1186/bcr2942 22114898 PMC3326547

[7] Vachon CM, van Gils CH, Sellers TA, Ghosh K, Pruthi S, Brandt KR, et al Mammographic density, breast cancer risk and risk prediction. Breast Cancer Res. 2007;9: 217 10.1186/bcr1829 18190724 PMC2246184

[8] Durduran T, Choe R, Baker WB, Yodh AG. Diffuse optics for tissue monitoring and tomography. Reports Prog Phys. 2010;73: 076701 (1–43). 10.1088/0034-4885/73/7/076701 PMC4482362 26120204

[9] Gibson AP, Hebden JC, Arridge SR. Recent advances in diffuse optical imaging. Phys Med Biol. 2005;50: 1–43. 10.1088/0031-9155/50/4/R01 15773619

[10] Gibson A, Dehghani H. Diffuse optical imaging. Philos Trans A Math Phys Eng Sci. 2009;367: 3055–72. 10.1098/rsta.2009.0080 19581255

[11] Shah N, Cerussi AE, Jakubowski D, Hsiang D, Butler J, Tromberg BJ. Spatial variations in optical and physiological properties of healthy breast tissue. J Biomed Opt. International Society for Optics and Photonics; 2004;9: 534–40. 10.1117/1.1695560 15189091

[12] Pogue BW, Jiang S, Dehghani H, Kogel C, Soho S, Srinivasan S, et al Characterization of hemoglobin, water, and NIR scattering in breast tissue: analysis of intersubject variability and menstrual cycle changes. J Biomed Opt. 2004;9: 541–52. 10.1117/1.1691028 15189092

[13] Shah N, Cerussi AE, Jakubowski D, Hsiang D, Butler J, Tromberg BJ. The role of diffuse optical spectroscopy in the clinical management of breast cancer. Dis Markers. 2004;19: 95–105. Available: http://www.ncbi.nlm.nih.gov/pubmed/15096707 10.1155/2004/460797 PMC3851626 15096707

[14] Wang X, Pogue BW, Jiang S, Song X, Paulsen KD, Kogel C, et al Approximation of Mie scattering parameters in near-infrared tomography of normal breast tissue in vivo. J Biomed Opt. 2005;10: 051704 10.1117/1.2098607 16292956

[15] Cubeddu R, Musolino M, Pifferi A, Taroni P, Valentini G. Time-resolved reflectance: a systematic study for application to the optical characterization of tissues. IEEE J Quantum Electron. 1994;30: 2421–2430. 10.1109/3.328616

[16] Pifferi A, Torricelli A, Taroni P, Comelli D, Bassi A, Cubeddu R. Fully automated time domain spectrometer for the absorption and scattering characterization of diffusive media. Rev Sci Instrum. AIP; 2007;78: 053103 10.1063/1.2735567 17552808

[17] Bassi A, Farina A, Andrea CD, Pifferi A, Valentini G, Cubeddu R. Portable, large-bandwidth time-resolved system for diffuse optical spectroscopy. Opt Express. 2007;15: 14482–14487. 19550726 10.1364/oe.15.014482

[18] Farina A, Bassi A, Pifferi A, Taroni P, Comelli D, Spinelli L, et al Bandpass effects in time-resolved diffuse spectroscopy. Appl Spectrosc. 2009;63: 48–56. 10.1366/000370209787169795 19146718

[19] Taroni P, Bassi A, Comelli D, Farina A, Cubeddu R, Pifferi A. Diffuse optical spectroscopy of breast tissue extended to 1100 nm. J Biomed Opt. International Society for Optics and Photonics; 2009;14: 054030 10.1117/1.3251051 19895132

[20] Mourant JR, Fuselier T, Boyer J, Johnson TM, Bigio IJ. Predictions and measurements of scattering and absorption over broad wavelength ranges in tissue phantoms. Appl Opt. 1997;36: 949–57. Available: http://www.ncbi.nlm.nih.gov/pubmed/18250760 18250760 10.1364/ao.36.000949

[21] Nilsson AM, Sturesson C, Liu DL, Andersson-Engels S. Changes in spectral shape of tissue optical properties in conjunction with laser-induced thermotherapy. Appl Opt. 1998;37: 1256–67. Available: http://www.ncbi.nlm.nih.gov/pubmed/18268713 18268713 10.1364/ao.37.001256

[22] Taroni P, Pifferi A, Quarto G, Spinelli L, Torricelli A, Abbate F, et al Noninvasive assessment of breast cancer risk using time-resolved diffuse optical spectroscopy. J Biomed Opt. 2010;15: 060501 10.1117/1.3506043 21198142

[23] Taroni P, Quarto G, Pifferi A, Ieva F, Paganoni AM, Abbate F, et al Optical identification of subjects at high risk for developing breast cancer. J Biomed Opt. International Society for Optics and Photonics; 2013;18: 060507 10.1117/1.JBO.18.6.060507 23804215

[24] Taroni P, Pifferi A, Salvagnini E, Spinelli L, Torricelli A, Cubeddu R. Seven-wavelength time-resolved optical mammography extending beyond 1000 nm for breast collagen quantification. Opt Express. OSA; 2009;17: 15932–46. 10.1364/OE.17.015932 19724592

[25] Patterson MS, Chance B, Wilson BC. Time resolved reflectance and transmittance for the non-invasive measurement of tissue optical properties. Appl Opt. Optical Society of America; 1989;28: 2331–6. 10.1364/AO.28.002331 20555520

[26] Haskell RC, Svaasand LO, Tsay TT, Feng TC, McAdams MS, Tromberg BJ. Boundary conditions for the diffusion equation in radiative transfer. J Opt Soc Am A Opt image Sci. 1994;11: 2727–41. Available: http://www.ncbi.nlm.nih.gov/pubmed/7931757 7931757 10.1364/josaa.11.002727

[27] D’Andrea C, Spinelli L, Bassi A, Giusto A, Contini D, Swartling J, et al Time-resolved spectrally constrained method for the quantification of chromophore concentrations and scattering parameters in diffusing media. Opt Express. 2006;14: 1888–98. Available: http://www.ncbi.nlm.nih.gov/pubmed/19503518 19503518 10.1364/oe.14.001888

[28] BI-RADS—Mammography 2013—American College of Radiology [Internet]. Available: http://www.acr.org/quality-safety/resources/birads/mammography. Accessed 2014 Jun 17.

[29] Stomper PC, D’Souza DJ, DiNitto PA, Arredondo MA. Analysis of parenchymal density on mammograms in 1353 women 25–79 years old. Am J Roentgenol. American Public Health Association; 1996;167: 1261–5. Available: http://www.ajronline.org/doi/abs/10.2214/ajr.167.5.8911192 8911192 10.2214/ajr.167.5.8911192

[30] Lee NA, Rusinek H, Weinreb J, Chandra R, Toth H, Singer C, et al Fatty and fibroglandular tissue volumes in the breasts of women 20–83 years old: comparison of X-ray mammography and computer-assisted MR imaging. AJR Am J Roentgenol. American Public Health Association; 1997;168: 501–6. 10.2214/ajr.168.2.9016235 9016235

[31] Boyd N, Martin L, Stone J, Little L, Minkin S, Yaffe M. A Longitudinal Study of the Effects of Menopause on Mammographic Features. Cancer Epidemiol Biomarkers Prev. 2002;11: 1048–1053. Available: http://cebp.aacrjournals.org/content/11/10/1048.long 12376506

[32] Checka CM, Chun JE, Schnabel FR, Lee J, Toth H. The relationship of mammographic density and age: implications for breast cancer screening. AJR Am J Roentgenol. American Roentgen Ray Society; 2012;198: W292–5. 10.2214/AJR.10.6049 22358028

[33] Li T, Sun L, Miller N, Nicklee T, Woo J, Hulse-Smith L, et al The association of measured breast tissue characteristics with mammographic density and other risk factors for breast cancer. Cancer Epidemiol Biomarkers Prev. 2005;14: 343–9. 10.1158/1055-9965.EPI-04-0490 15734956

[34] King V, Gu Y, Kaplan JB, Brooks JD, Pike MC, Morris EA. Impact of menopausal status on background parenchymal enhancement and fibroglandular tissue on breast MRI. Eur Radiol. 2012;22: 2641–7. 10.1007/s00330-012-2553-8 22752463

[35] Pasarica M, Sereda OR, Redman LM, Albarado DC, Hymel DT, Roan LE, et al Reduced Adipose Tissue Oxygenation in Human Obesity: Evidence for Rarefaction, Macrophage Chemotaxis, and Inflammation Without an Angiogenic Response. Diabetes. 2008;58: 718–725. 10.2337/db08-1098 19074987 PMC2646071

[36] Dorgan JF, Klifa C, Shepherd JA, Egleston BL, Kwiterovich PO, Himes JH, et al Height, adiposity and body fat distribution and breast density in young women. Breast Cancer Res. 2012;14: R107 10.1186/bcr3228 22800711 PMC3680938

[37] Boyd N, Martin L, Chavez S, Gunasekara A, Salleh A, Melnichouk O, et al Breast-tissue composition and other risk factors for breast cancer in young women: a cross-sectional study. Lancet Oncol. 2009;10: 569–80. 10.1016/S1470-2045(09)70078-6 19409844

[38] Isaksson E, von Schoultz E, Odlind V, Söderqvist G, Csemiczky G, Carlström K, et al Effects of Oral Contraceptives on Breast Epithelial Proliferation. Breast Cancer Res Treat. 2001;65: 163–169. 10.1023/A:1006482418082 11261832

[39] Anderson GL, Autier P, Beral V, Bosland MC, Fernandez E, Haslam SZ, et al. IWG on the E of CR to H. Combined Estrogen-progestogen Contraceptives and Combined Estrogen-progestogen Menopausal Therapy [Internet]. Lyon; 2007. Available: http://books.google.com/books?hl=it&lr=&id=aGDU5xibtNgC&pgis=1

[40] Ueda S, Nakamiya N, Matsuura K, Shigekawa T, Sano H, Hirokawa E, et al Optical imaging of tumor vascularity associated with proliferation and glucose metabolism in early breast cancer: clinical application of total hemoglobin measurements in the breast. BMC Cancer. 2013;13: 514 10.1186/1471-2407-13-514 24176197 PMC3817816

[41] Provenzano PP, Eliceiri KW, Campbell JM, Inman DR, White JG, Keely PJ. Collagen reorganization at the tumor-stromal interface facilitates local invasion. BMC Med. 2006;4: 38 10.1186/1741-7015-4-38 17190588 PMC1781458

[42] Barcus CE, Keely PJ, Eliceiri KW, Schuler L a. Stiff collagen matrices increase tumorigenic prolactin signaling in breast cancer cells. J Biol Chem. 2013;288: 12722–32. 10.1074/jbc.M112.447631 23530035 PMC3642318

[43] Luparello C. Aspects of Collagen Changes in Breast Cancer. J Carcinog Mutagen. 2013;S13 10.4172/2157-2518.S13-007

[44] Boyd NF, Li Q, Melnichouk O, Huszti E, Martin LJ, Gunasekara A, et al Evidence that breast tissue stiffness is associated with risk of breast cancer. PLoS One. Public Library of Science; 2014;9: e100937 10.1371/journal.pone.0100937 25010427 PMC4091939

[45] Pike MC, Pearce CL. Mammographic density, MRI background parenchymal enhancement and breast cancer risk. Ann Oncol. 2013;24 Suppl 8: viii37–viii41. 10.1093/annonc/mdt310 24131968 PMC3894109

[46] Choe R, Durduran T. Diffuse Optical Monitoring of the Neoadjuvant Breast Cancer Therapy. IEEE J Sel Top Quantum Electron. 2012;18: 1367–1386. 10.1109/JSTQE.2011.2177963 23243386 PMC3521564

[47] Tromberg B, L’Heureux D, Mankoff D, Zhang Z, Cerussi A, Mehta R, et al OT2-05-02: ACRIN 6691 Monitoring and Predicting Breast Cancer Neoadjuvant Chemotherapy Response Using Diffuse Optical Spectroscopic Imaging (DOSI). Cancer Res. 2012;71: OT2–05–02–OT2–05–02. 10.1158/0008-5472.SABCS11-OT2-05-02

[48] Hattangadi J, Park C, Rembert J, Klifa C, Hwang J, Gibbs J, et al Breast stromal enhancement on MRI is associated with response to neoadjuvant chemotherapy. AJR Am J Roentgenol. American Roentgen Ray Society; 2008;190: 1630–6. 10.2214/AJR.07.2533 18492917

[49] Evans A, Armstrong S, Whelehan P, Thomson K, Rauchhaus P, Purdie C, et al Can shear-wave elastography predict response to neoadjuvant chemotherapy in women with invasive breast cancer? Br J Cancer. 2013;109: 2798–802. 10.1038/bjc.2013.660 24169359 PMC3844913

[50] Kim J, Han W, Moon H-G, Ahn S, Shin H-C, You J-M, et al Breast density change as a predictive surrogate for response to adjuvant endocrine therapy in hormone receptor positive breast cancer. Breast Cancer Res. 2012;14: R102 10.1186/bcr3221 22770227 PMC3680951

