# Supplementary material for: Breast Tissue Composition and Its Dependence on Demographic Risk Factors for Breast Cancer: Non-Invasive Assessment by Time Domain Diffuse Optical Spectroscopy
Source: PLoS One. 2015 Jun 1;10(6):e0128941. doi: 10.1371/journal.pone.0128941 (PMC4452361; doi:10.1371/journal.pone.0128941)
Supplement: S1 File — Demographic information and optically derived parameters. (PDF) [file pone.0128941.s001.pdf]

				1=pre 2=post 3=NA	1=yes 2=no 3=NA	1,1=current 1,2=past 1,3=NA								
**Patient ID**	**Age (y)**	**Weight (Kg)**	**Height (cm)**	**Menopausal status**	**OC use**	**Current OC use**	**BI-RADS**	**a (cm-1)**	**b**	**tHb (uM)**	**SO2 (%)**	**Lipid (mg/cm3)**	**Water (mg/cm3)**	**Collagen (mg/cm3)**
1	44	59	160	1	2		3	20.72	0.74	9.06	61.73	447.56	460.78	119.90
2	63	70	160	2	2		1	9.99	0.34	8.76	91.91	802.29	64.57	58.71
3	75	82	165	2	2		1	13.08	0.43	10.82	79.25	778.43	118.71	40.51
4	55	68	163	3	2		2	12.43	0.53	10.78	87.81	761.21	97.76	38.24
5	45	49	158	1	2		3	15.70	0.76	12.22	89.60	653.62	192.22	54.04
6	49	61	153	2	1	1,2	2	13.88	0.40	9.93	89.72	676.50	124.98	105.20
7	43	70	168	1	1	1,2	1	11.14	0.62	9.07	86.73	716.17	79.12	61.00
8	48	65	160	2	2		3	9.74	0.77	10.44	74.35	648.92	217.25	49.23
9	40	59	164	1	2		3	14.71	1.01	11.53	90.66	676.79	135.52	52.85
10	64	64	170	2	1	1,2	2	14.71	0.42	7.79	94.79	665.17	118.52	64.13
11	43	69	166	2	1	1,2	3	6.53	1.44	14.34	67.76	666.17	156.39	54.05
12	78	45	150	2	2			15.31	0.51	12.53	99.05	582.59	144.69	77.40
13	40	105	172	1	1	1,2	2	11.82	0.40	6.80	80.82	791.10	72.15	43.13
14	56	52	150	2	2		2	10.53	0.50	9.67	85.71	802.01	68.40	36.02
15	47	50	161	1	1	1,2	4	18.37	1.09	3.88	79.00	310.35	672.92	114.00
16	41	62	173	1	2		3	19.27	0.83	12.22	97.52	502.07	406.22	136.51
17	57	74	168	2	2		2	11.46	0.49	9.88	88.22	721.46	114.45	46.54
18	61	67	165	2	2			13.73	0.41	9.48	82.37	725.30	113.11	79.32
19	45	58	163	2	2		3	13.03	0.75	8.35	74.87	676.48	144.15	68.07
20	55	57	160	2	2		2	13.66	0.52	8.62	88.60	702.18	104.41	63.80
21	47	54	162	1	2		3	14.76	0.54	10.49	92.86	632.41	199.39	119.79
22	72	73	156	2	2		1	11.40	0.43	8.63	80.80	829.04	75.02	40.91
23	65	71	164	2	2		2	11.28	0.48	8.83	91.65	739.16	93.14	60.28
24	39	51	160	1	1	1,2	4	18.07	1.08	12.87	99.65	379.18	426.53	122.60
25	36	46	167	1	1	1,2	4	17.56	1.14	13.99	97.95	364.91	608.60	113.86
26	44	78	163	2	1	1,2	2	10.90	0.47	8.89	90.77	806.65	76.84	42.11
27	54	63	160	2	2		2	12.48	0.51	11.62	96.67	662.89	88.08	60.06
28	46	49	165	1	2		4	16.32	0.70	12.49	92.93	420.62	490.92	179.67
29	53	63	165	2	2		3	16.29	0.67	8.81	83.27	600.87	236.47	68.02
30	68	72	165	2	2		3	12.96	0.61	14.76	90.80	666.15	137.81	67.55
31	36	55	160	1	2		4	18.19	0.91	12.07	98.64	361.24	563.31	171.10
32	58	62	158	2	1	1,2	1	11.71	0.44	7.17	79.83	775.10	78.63	27.82
33	46	48	156	1	1	1,2	2	17.02	0.69	16.60	89.89	572.70	293.89	60.63
34	41	48	155	2	1	1,2	2	8.84	0.64	14.23	91.69	598.92	168.14	69.44
35	68	48	162	2	2		2	13.25	0.50	12.92	91.82	697.03	120.45	51.96
36	63	55	160	2	2		3	17.58	0.55	11.07	84.70	578.76	266.09	65.03
37	48	47	155	1	1	1,2	3	14.48	0.64	13.60	93.98	551.91	236.80	92.71
38	38	43	160	1	1	1,2	4	14.70	0.92	16.83	91.78	452.82	445.82	128.32
39	32	61	178	1	2			14.96	0.75	14.73	94.09	597.89	247.62	105.58
40	41	58	155	1	2		2	11.41	0.34	10.76	92.99	759.48	84.73	45.16

Patient ID	Age (y)	Weight (Kg)	Height (cm)	Menopausal status<br>1=pre<br>2=post<br>3=NA	OC use<br>1=yes<br>2=no<br>3=NA	Current OC use<br>1,1=current<br>1,2=past<br>1,3=NA	BI-RADS	a (cm-1)	b	tHb (uM)	SO2 (%)	Lipid (mg/cm3)	Water (mg/cm3)	Collagen (mg/cm3)
41	31	57	170	1	1	1,1		18.35	1.03	13.28	90.54	381.83	597.22	143.50
42	65	80	167	2	2		2	11.32	0.44	8.40	86.31	845.16	72.32	47.13
43	54	68	160	2	1	1,2	2	10.14	0.39	8.99	85.56	801.80	79.06	38.81
44	55	68	160	2	1	1,2	3	9.57	1.13	10.64	78.70	703.41	143.19	51.38
45	62	67	163	2	1	1,2	2	10.28	0.46	10.17	93.64	777.99	77.39	50.90
46	66	80	167	2	2		1	11.80	0.31	7.47	92.58	757.30	55.41	62.52
47	53	53	164	2	1	1,2	2	13.50	0.41	16.98	91.83	721.35	125.50	51.76
48	51	48	167	1	2		3	19.62	0.54	18.29	92.85	529.81	300.68	146.26
49	55	64	168	2	1	1,2	2	10.28	0.51	9.27	90.62	817.33	65.26	44.22
50	68	70	164	2	2		3	12.37	0.62	9.56	92.41	643.94	136.03	71.12
51	39	60	159	1	2		4	16.36	1.04	12.57	84.43	554.10	295.13	75.38
52	41	70	169	1	2		4	10.72	1.61	10.71	73.67	606.69	257.24	50.63
53	40	52	165	2	1	1,2	3	16.06	0.51	10.48	92.00	624.52	207.50	129.53
54	51	58	150	1	1	1,1	2	12.32	0.44	8.50	89.19	766.94	91.29	39.23
55	78	73	158	2	2		1	11.43	0.49	6.24	77.12	819.20	69.99	22.52
56	36	55	158	1	1	1,1	4	14.36	1.05	16.90	95.15	532.36	390.32	125.81
57	50	59	162	2	1	1,2	2	16.99	0.60	7.38	72.66	594.22	175.86	122.35
58	54	50	156	2	2		3	16.19	0.43	14.71	97.99	646.78	142.61	113.89
59	67	67	165	2	1	1,2	3	12.55	0.53	7.81	74.28	785.70	96.45	63.60
60	47	59	165	1	1	1,2	3	12.73	0.42	16.44	99.93	599.04	124.27	115.70
61	59	53	157	2	1	1,2		13.15	0.59	8.99	87.92	705.37	143.49	65.37
62	79	63	165	2	2		1	10.80	0.18	7.44	96.64	744.26	65.48	83.45
63	48	70	162	1	2		1	10.65	0.43	9.33	85.78	817.66	67.62	34.31
64	75	61	155	2	1	1,2	1	11.87	0.40	10.78	89.83	758.44	83.66	37.98
65	53	63	154	2	1	1,2	2	9.59	0.43	9.21	85.32	811.20	65.66	36.05
66	43	84	175	1	2		1	11.83	0.33	10.21	91.62	738.66	92.54	44.94
67	61	50	155	2	1	1,2	1	10.48	0.37	9.77	87.10	788.70	69.22	30.82
68	58	70	170	2	1	1,2	2	10.24	0.44	12.19	87.24	853.73	68.56	30.35
69	37	51	158	1	2		3	18.67	0.77	18.72	99.78	337.56	416.35	188.23
70	50	59	160	1	1	1,2	2	12.41	0.49	8.86	88.49	732.15	115.25	57.37
71	71	93	165	2	2			11.11	0.46	11.31	87.36	811.78	74.14	50.13
72	60	80	160	2	2			10.22	0.40	10.38	80.65	779.81	65.10	41.09
73	41	50	164	2	1	1,2	3	19.74	0.91	10.31	93.15	495.43	457.33	83.64
74	68	48	150	2	2		1	12.25	0.46	11.68	90.67	737.08	99.92	52.62
75	47	80	160	3	1	1,2		10.96	0.54	10.38	89.82	735.08	87.87	64.97
76	66	80	165	2	1	1,2	1	10.19	0.30	10.19	97.69	703.86	65.49	68.21
77	45	52	164	1	2		1	12.15	0.53	7.57	91.08	741.55	77.47	47.13
78	40	55	163	1	1	1,2		16.27	0.81	12.65	90.53	521.34	353.67	96.27
79	47	60	167	1	1	1,2	3	13.47	0.53	12.08	98.18	577.28	108.73	97.42
80	45	48	168	1	1	1,1	4	17.45	1.09	16.02	93.94	614.46	196.37	113.74

				1=pre 2=post 3=NA	1=yes 2=no 3=NA	1,1=current 1,2=past 1,3=NA								
**Patient ID**	**Age (y)**	**Weight (Kg)**	**Height (cm)**	**Menopausal status**	**OC use**	**Current OC use**	**BI-RADS**	**a (cm-1)**	**b**	**tHb (uM)**	**SO2 (%)**	**Lipid (mg/cm3)**	**Water (mg/cm3)**	**Collagen (mg/cm3)**
81	50	59	164	2	1	1,2		12.53	0.51	13.40	90.12	653.23	115.80	62.76
82	59	80	162	2	2			9.89	0.47	9.52	80.77	881.33	70.57	23.60
83	61	80	163	2	2		1	11.10	0.63	7.43	68.88	866.88	82.14	14.91
84	68	65	160	2	2			11.44	0.45	14.30	84.36	822.31	86.35	32.29
85	51	48	160	2	2		4	15.60	0.50	27.86	98.10	286.77	447.98	224.00
86	66	46	152	2	2		1	14.07	0.45	21.41	87.40	696.05	168.88	48.15
87	35	63	160	1	1	1,2	4	16.95	1.04	13.18	88.59	577.76	266.61	63.78
88	55	62	170	2	1	1,2	3	16.66	0.61	15.92	92.27	506.26	248.61	164.68
89	56	58	158	2	1	1,2	3	11.66	0.83	8.89	76.02	730.91	98.61	66.40
90	45	63	157	1	2		4	15.91	0.89	13.17	80.19	624.16	235.08	88.21
91	79	55	160	2	2		2	13.79	0.40	10.89	79.36	745.83	122.79	70.79
92	44	81	160	1	1	1,2		11.13	1.01	8.45	64.72	708.07	76.63	57.12
93	66	65	165	2	2		4	13.88	0.84	11.10	84.66	684.32	181.47	50.41
94	43	76	176	1	1		3	15.41	0.42	16.10	84.99	694.35	223.53	87.26
95	71	65	158	2	2		2	11.10	0.30	9.98	89.45	764.01	102.71	51.38
96	45	71	158	1	2		3	10.02	0.75	10.22	70.51	742.39	96.46	30.65
97	62	107	180	2	2		1	10.79	0.89	11.45	78.28	783.37	58.83	45.11
98	65	60	150	2	2		2	11.15	0.44	6.84	94.71	857.15	76.43	75.16
99	51	72	169	1	1	1,1	2	12.65	0.39	13.99	94.80	811.11	133.63	79.55
100	53	58	157	1	2		4	14.04	0.84	17.75	87.34	645.37	202.45	108.92
101	65	82	155	2	2			9.86	0.27	9.04	93.29	825.78	70.31	94.38
102	43	72	158	1	1	1,1	3	12.23	0.38	7.67	80.44	791.90	125.57	54.66
103	54	54	170	2	1	1,2	3	17.54	0.49	17.00	98.45	429.75	296.01	204.30
104	64	75	170	2	1	1,2		12.00	0.43	10.30	82.57	846.10	69.29	32.16
105	58	57	163	2	1	1,2	3	10.84	0.45	10.30	93.81	710.47	114.47	104.62
106	50	62	164	1	1	1,2	4	17.62	0.70	16.72	99.79	469.17	292.42	152.49
107	59	68	164	2	2		3	12.98	0.89	10.22	68.16	782.83	106.92	66.42
108	67	64	165	2	2		2	12.38	0.33	9.80	93.09	794.30	102.26	55.82
109	47	61	157	2	2		4	12.48	1.68	12.02	61.66	531.05	82.65	53.30
110	54	72	166	1	1	1,1	3	11.32	0.54	13.22	93.38	722.03	74.84	67.58
111	36	51	165	1	1	1,1	3	16.97	0.47	21.47	93.14	470.98	363.85	162.48
112	54	80	175	2	1	1,2	3	12.34	0.36	5.59	84.71	762.41	123.68	80.60
113	79	69	168	2	2		2	12.23	0.50	4.80	67.92	794.20	106.99	39.42
114	44	65	167	1	2		4	14.54	1.63	9.47	56.42	478.07	165.64	63.62
115	41	48	155	1	1	1,2	4	12.50	0.76	13.02	89.27	649.78	229.41	86.98
116	35	53	169	1	1	1,3	3	18.46	0.83	22.87	71.80	269.95	537.38	198.92
117	77	77	165	2	3		2	10.14	0.33	8.78	89.44	804.43	124.06	48.72
118	62	58	148	2	2		1	10.68	0.39	9.57	85.20	801.58	91.33	42.63
119	54	85	164	1	1	1,2	4	13.28	0.53	11.23	94.98	664.82	128.86	112.35
120	46	55	168	1	2		4	16.54	0.78	18.84	85.93	282.04	472.87	142.96

Patient ID	Age (y)	Weight (Kg)	Height (cm)	1=pre 2=post 3=NA Menopausal status	1=yes 2=no 3=NA OC use	1,1=current 1,2=past 1,3=NA Current OC use	BI-RADS	a (cm-1)	b	tHb (uM)	SO2 (%)	Lipid (mg/cm3)	Water (mg/cm3)	Collagen (mg/cm3)
121	72	80	168	2	2			11.54	0.43	10.50	84.80	719.42	105.88	49.78
122	78	56	165	2	2		4	13.44	0.51	8.55	70.54	668.45	170.01	77.94
123	62	66	160	2	2		3	10.41	0.45	8.78	82.29	821.38	89.42	38.21
124	53	80	158	1	2		3	9.09	0.40	10.31	90.64	826.72	86.35	55.16
125	45	70	168	1	2		3	13.15	0.49	9.92	87.29	787.11	102.06	66.45
126	44	52	168	1	1	1,3	4	15.19	0.53	15.74	86.20	521.53	366.70	134.04
127	78	63	158	2	2		2	11.42	0.42	7.95	77.08	870.27	74.80	34.54
128	61	64	154	2	1		2	10.19	0.38	6.90	86.34	821.23	84.45	40.72
129	33	60	156	1	2			12.95	0.72	11.25	91.08	665.39	192.77	79.38
130	40	58	162	1	2		4	16.36	0.83	7.59	80.85	485.93	469.94	105.61
131	43	64	164	1	1	1,1	3	13.73	0.48	16.69	92.58	666.27	227.06	106.67
132	55	60	160	2	2		3	12.39	0.45	6.34	82.50	769.63	120.49	74.65
133	50	46	150	2	2		4	18.30	0.85	8.24	88.15	547.96	254.07	104.88
134	55	67	165	2	2			10.72	0.39	9.62	80.98	909.66	61.58	31.49
135	75	73	160	2	2		2	12.00	0.27	8.18	78.81	827.63	101.26	31.47
136	56	57	160	2	2		3	12.69	0.52	9.29	79.49	764.47	105.74	66.00
137	45	75	165	1	1	1,2	3	11.73	0.50	6.64	74.55	824.96	91.58	45.74
138	39	60	167	1	2		3	11.97	0.92	7.13	48.57	665.28	173.95	51.36
139	55	62	160	2	2		4	14.46	0.82	11.25	66.23	628.97	203.16	82.86
140	46	50	160	1	1	1,3	3	18.47	0.33	18.74	83.97	567.87	325.16	159.86
141	42	56	168	2	1	1,2	4	18.63	1.26	10.39	73.44	342.84	588.96	94.83
142	33	70	172	1	1	1,1		12.98	0.83	15.14	93.67	595.25	222.37	112.25
143	19	51	170	1	3			15.97	1.26	17.36	98.70	802.98	120.74	121.91
144	43	60	170	1	1	1,2	3	13.60	0.63	16.00	91.86	650.26	216.16	91.57
145	30	73	174	1	2			12.01	0.81	8.76	86.98	685.30	134.83	72.27
146	56	55	167	2	2		4	20.08	0.56	7.92	54.23	592.19	314.91	85.86
147	43	59	170	1	1	1,2	3	16.36	0.44	11.70	84.56	542.93	328.88	146.28
148	43	50	160	1	1	1,2		15.50	0.62	13.16	94.91	479.70	361.27	151.39
149	54	85	165	2	2		3	11.91	0.61	7.84	83.90	771.23	100.93	53.46
150	39	50	160	1	2			17.39	0.95	16.39	67.28	400.56	558.72	110.60
151	35	60	172	1	1	1,2	4	16.84	0.96	17.53	76.44	540.87	382.14	59.30
152	44	59	174	1	2		3	16.31	0.67	17.34	84.70	526.17	310.69	74.49
153	65	62	155	2	2			9.80	0.32	8.62	83.54	857.76	69.22	44.98
154	48	63	165	1	1	1,2	3	12.63	0.37	12.76	83.47	733.71	187.25	61.77
155	43	68	168	1	2		3	13.29	0.44	10.48	93.25	636.58	194.29	101.44
156	44	63	158	1	2			13.44	0.37	13.50	88.46	696.73	190.43	82.72
157	53	80	172	2	2		3	11.21	0.66	10.15	84.51	781.97	74.64	49.98
158	38	49	167	1	1	1,2	4	17.39	0.68	22.81	91.53	449.46	326.06	220.16
159	67	52	158	2	2			17.81	0.40	16.90	71.92	610.65	284.87	125.25
160	60	68	163	2	2		2	10.64	0.38	12.96	89.42	809.75	93.69	50.48

				1=pre 2=post 3=NA	1=yes 2=no 3=NA	1,1=current 1,2=past 1,3=NA								
Patient ID	Age (y)	Weight (Kg)	Height (cm)	Menopausal status	OC use	Current OC use	BI-RADS	a (cm-1)	b	tHb (uM)	SO2 (%)	Lipid (mg/cm3)	Water (mg/cm3)	Collagen (mg/cm3)
161	51	48	164	1	2		4	19.96	0.53	14.96	93.91	440.11	422.95	143.65
162	41	60	160	1	1	1,2	2	18.20	0.40	18.75	74.29	690.71	229.72	75.74
163	45	53	160	1	2		3	17.17	0.37	13.23	72.92	699.17	212.06	48.48
164	59	56	162	2	1	1,2	4	17.47	0.50	9.40	99.07	648.60	178.23	134.38
165	43	63	160	1	1	1,2		15.10	0.52	12.18	95.27	667.82	275.05	97.89
166	40	60	172	1	1	1,1	4	15.53	1.21	15.02	75.46	587.69	261.95	93.62
167	47	65	158	3	2		3	12.34	0.63	12.61	74.24	734.46	104.00	43.64
168	43	69	164	1	2		3	16.87	0.77	14.27	84.67	631.17	300.43	88.55
169	39	53	165	1	1	1,2	4	15.06	1.02	16.89	87.16	614.63	261.20	75.07
170	45	45	158	1	1	1,2	3	17.50	0.40	12.11	87.16	568.55	331.84	230.92
171	39	47	160	1	1	1,2	4	11.84	1.11	22.42	79.04	583.08	150.25	91.42
172	57	66	165	2	1	1,2	2	10.67	1.04	7.31	60.15	805.93	66.34	37.60
173	46	50	160	1	1	1,2	3	15.62	0.56	22.02	91.28	587.32	341.75	129.83
174	46	50	157	3	1	1,2		18.37	0.88	11.21	85.39	519.61	331.09	128.73
175	50	65	160	3	2			13.18	0.38	7.45	81.22	794.19	125.06	54.73
176	53	61	147	2	2			9.52	0.43	7.51	80.78	901.32	56.36	47.43
177	61	63	160	2	2			12.69	0.42	13.40	83.98	785.34	110.58	51.46
178	64	69	162	2	2			11.60	0.30	7.62	97.05	782.43	96.44	93.37
179	48	52	158	1	2		3	13.55	0.77	12.74	84.73	726.67	163.85	43.85
180	54	64	160	2	1	1,2		11.21	0.41	7.61	84.34	827.01	95.11	57.47
181	49	60	170	1	2			15.42	0.52	13.11	94.01	630.89	194.66	108.11
182	50	60	158	1	1	1,2		12.19	0.59	18.24	87.18	711.26	185.50	113.05
183	43	50	165	1	2			18.39	0.88	12.63	80.44	493.53	429.03	84.15
184	43	50	160	1	2			16.46	0.84	17.15	86.00	528.24	394.98	118.66
185	67	88	170	2	2			12.12	0.33	12.18	91.77	756.54	104.17	75.01
186	46	52	160	1	2			15.00	0.81	25.34	94.23	359.43	630.95	173.62
187	50	59	160	1	2			13.60	0.49	12.53	90.74	766.72	130.99	49.42
188	60	62	165	2	3			9.74	0.71	10.89	84.16	794.35	89.82	35.37
189	48	58	165	1	2			15.03	0.53	16.79	85.53	694.63	207.21	107.32
190	40	54	160	1	1	1,1		16.10	0.88	15.53	79.49	544.00	335.64	94.54
191	52	86	168	3	1	1,3		10.79	0.40	8.64	90.29	793.91	83.58	60.95
193	73	56	150	2	2			12.52	0.36	11.24	87.08	784.54	97.75	45.10
194	58	69	167	1	1	1,2		10.25	0.35	10.20	93.73	736.95	69.26	86.36
195	63	72	160	2	2			10.40	0.46	11.50	85.61	837.02	59.36	38.55
196	40	62	159	1	1	1,2		11.31	0.70	14.81	75.92	618.53	154.40	106.85
197	60	56	156	2	1	1,2		14.47	0.40	10.79	97.86	691.73	143.74	94.44
198	66	53	156	2	2			12.59	0.41	14.25	92.71	744.76	125.80	93.78
199	78	55	150	2	2			12.25	0.59	11.27	78.95	762.12	114.54	50.70
200	32	58	171	1	1	1,2		14.93	0.62	13.64	97.19	643.62	177.25	113.63
201	39	53	154	1	1	1,2		14.43	0.57	11.65	91.93	725.15	165.01	65.92
